# Pregnancy Planning and Genetic Testing: Exploring Advantages, and Challenges

**DOI:** 10.3390/genes15091205

**Published:** 2024-09-13

**Authors:** Ulf Kristoffersson, Maria Johansson-Soller

**Affiliations:** 1 Division of Clinical Genetics, Department of Laboratory Medicine, Lund University, 22100 Lund, Sweden; 2 Department of Clinical Genetics, Karolinska University Hospital, 17176 Solna, Sweden; maria.johansson-soller@regionstockholm.se

**Keywords:** pregnancy planning and genetic testing, preconception genetic testing, genetic disorders, reproductive healthcare, genetic counselling, ethical and societal considerations

## Abstract

Pregnancy planning and genetic testing (PPGT) has emerged as a tool in reproductive healthcare, offering parents-to-be insight in their risks of having a child with a genetic disorder. This paper reviews the advantages, drawbacks and challenges associated with PPGT, providing some practical guidance for health care professionals. Advantages include identification of genetic risks, a possibility to informed reproductive decision-making, and the potential to reduce the parents-to-be risk for an affected child. Challenges and drawbacks include provision of service, ethical considerations, genetic counselling complexities, and the need to increase public and professional awareness by comprehensive education and accessibility. Practical guidance involves considerations for selecting appropriate candidates, counselling strategies, and how to integrate PPGT into existing healthcare frameworks. By addressing these factors, PPGT can offer an increased reproductive informed choice for the individual and the couple reducing the burden of disease in the family.

## 1. Introduction

The advancement of molecular genetic technology over the last decade has enabled a wider use for genetic testing both inside the health care systems for diagnostic or treatment purposes, for cascade testing and presymptomatic testing in families, or outside, e.g., for ancestry testing or legal purposes. As a part of this extended use of molecular genetic technologies testing of individuals without previously known risk has emerged as a possibility. Thus, an increased use for pregnancy planning has appeared outside the health care system. Private laboratories offer now tests to couples who want to find out whether they are carriers of a pathogenic genetic variant in the same gene. 

This preconception genetic testing (PPGT) enables individuals or couples to assess their risk for passing pathogenic genetic variants associated with severe early onset recessive or X-linked disorders to their offspring prior to conception, avoiding giving birth to a disabled child or abortion. Prenatal diagnosis via chorionic villi biopsy or amniocentesis does not give the same opportunities to assess pathogenic genetic variation due to time constraint and limited possibilities to apply multigene testing. Even if it in the future would become possible to assess multiple genetic conditions, during early pregnancy, it would be associated with offering complex information and a burdensome to the parents-to-be about a previously unknown conditions in the family which in turn may lead to interruption of the pregnancy. 

Thus, PPGT offers today an opportunity for individuals or parents-to-be to gain knowledge of their genetic profiles before a pregnancy enabling informed reproductive decisions based on their genetic profiles. The possibilities to use PPGT has expanded rapidly in recent years, driven by advances in molecular genetic technologies and increased awareness of genetic disease risks among lay people [1].

Therefore, general practitioners as well as non-genetic specialists may meet patients who have done tests themselves or have relatives who did, or who want help to be tested. This paper is aimed to try to guide non-genetic specialists who might meet patients interested in preconception testing [2,3]

It will discuss the advantages and possibilities associated with PPGT as well as challenges and address some of the ethical concerns, also adding an attempt to help medical healthcare professionals to assist their patients to an informed decision regarding PPGT.

The focus of the discussion is PPGT in the non-consanguineous situation, as special considerations for selection of tested genes must be taken if you have particular founder mutations in the population. Similar considerations need to be taken when consanguineous couples ask for PPGT.

## 2. Search Strategy and Screening of Literature

The PubMed database was searched for full-text articles discussing different aspects of preconception carrier screening in August 2024. Different combinations of search terms relating to preconception carrier screening; reproductive genetic screening, extended carrier screening, expanded carrier screening and terms autonomy, ethics, socioeconomic, and ethnical was used to cover the topic as careful as possible. 

We have collected 680 potentially eligible studies to assess for inclusion after duplicates were removed. Studies not meeting our search criteria were excluded leaving 404 articles for further evaluation. 

Exclusion criteria were:

- Articles published before 2015, except regarding ethical issues
- Articles describing testing of single disorders
- Use of older methods than DNA-panels
- Used in third part screening or IVF/PGT or prenatal screening
- Not in English
- Not an original research article

After evaluation and forward/backward citation searching 83 studies were found eligible for inclusion. 

## 3. Strategies for PPGT

The primary goal of carrier screening is to identify asymptomatic individuals who carry variants associated with genetic diseases, to inform about future own risk, or risk to have a child with a genetic disease. Carrier screening can be accomplished through different approaches including ethnicity-based screening, pan-ethnic screening, and expanded carrier screening (ECS). The decision to perform carrier screening is voluntary. ECS takes a broad approach by screening for many genetic diseases irrespective of ethnic background, and ideally is performed prior to conception. ECS has many benefits, including that it does not depend on accuracy of reported ancestry, as well as it increases the yield of information that may be used for reproductive decision-making. However, there are also several important limitations of ECS to consider, ranging from the yield of unexpected information, uncertainty about the phenotype of a particular disease for which an individual is a carrier. Further, on a societal level, increasing costs associated with family testing and genetic counselling have to be considered. Detailed genetic counselling both prior to and after ECS is essential in order for patients to understand the breadth of this approach, potential and actual results, and limitations.

Every person is a carrier of at least a handful of pathogenic variants, mostly ultrarare, in genes that are poorly studied and with scarce knowledge of gene/disease association making classification of the individual variants difficult. Many of these genes are not affecting quality of life but may predict for future disorders in adult life or senescence. Most studies on prenatal or preimplantation genetic testing are focused on rare disorders affecting early in life with no available curative treatment [4].

For PPGT, testing is therefore recommended to be limited to autosomal or X-linked disorders associated with severe early onset where no curable treatment exists or with severely disabling malformations.

It is not possible to delineate in absolute terms what a severe genetic disorder is. It is in part observer dependent, being the affected person, the caretaker, the medical professional, etc. An attempt to define a severe disorder has been performed. Lazarin et al. tried to evaluate severity based on 15 criteria in 4 tiers [5]. They sent a questionnaire to a large number of health professionals, mainly medical genetics staff, and with help of the answers they constructed a four-step scale, profound, severe, moderate and mild. This scale can be used as a tool to help in the evaluation of how suitable prenatal or preconception genetic testing could be for an individual disorder or genetic variant. However, still all determinants on what consists a severe genetic disease remains subjective. Therefore, it is impossible to give a one-for-all definition of the severity of a rare genetic disorder. 

Further, for many genes the pathogenic variants may show reduced penetrance and/or variable expressivity for the specific disease further complicating presymptomatic and prenatal testing. Therefore, the recommendations are to limit the number of tested genes to the most common and well-studied of the rare disease genes, and to those variants that can be satisfactory classified as disease associated rather than including as many genes as possible [6,7].

Any individual or couple planning for a pregnancy can have utility of PPGT. Testing can be performed for a single person who want to know about his/her genetic profile of recessive and for women also X-linked pathogenic variants. However, this testing is usually not included in health insurance programmes and therefore not affordable to all. Inclusion in health care programmes are, if existing, limited to target populations with a high risk for carriership of specific disorders. If in the future, included in health care programmes, equal opportunity to testing is of great importance for non-discrimination, which have been pointed out in several studies [8].

Knowledge of being a carrier may seem unimportant as it usually does not have any impact on their own health. However, for certain conditions such as haemophilia or FragileX are associated with a vulnerability for clinical symptoms and disease. Both disorders are X-linked, and female haemophilia pathogenic variant carriers may have an increased bleeding risk due to skewed X-chromosome inactivation. Female carriers of a premutation, 55-200 CCG repeats in the FragileX causing *FMR1*-gene may develop premature menopause and males a Parkinson-like movement disorder. Thus, knowing this could be an advantage as preventive actions can be taken for the carrier. 

Even in those cases where the carrier do not have any symptoms of disease, carriership can be troublesome or even a burden. Knowledge of future risks in the family planning process can affect the choice of a partner, and even if preventive measures such as different forms of prenatal diagnosis are available, it may conflict with personal beliefs.

An alternative approach could be to only offer couple testing, i.e., only a pair planning a pregnancy are tested and only when both are carriers of pathogenic variants in the same gene, information about the variants is disclosed. The burden of knowledge is then associated to an individual disorder with 25 percent risk for the offspring, and thus more associated with the reproductive process such as prenatal diagnosis or preimplantation embryo selection. To avoid that the female partners carrier status is disclosed this approach doesn’t include testing for X-linked disorders [9,10]. 

In principle, population screening with whole genome sequencing will give an opportunity to offer testing to all individuals in an unselected population to reveal the burden of genetic disease and to offer possibilities for prevention. However, in spite of the enormous increase of knowledge about the human genome, very little is known about specific gene—disease interactions. For less than 200 genes there are relevant knowledge to understand the association between the gene variant and disease. However, new ultrarare variants are popping up at each sequencing of the human genome, but the gene-disease association is difficult to assess in the absence of data from more than a single individual. Furthermore, we have little knowledge about penetrance and expressivity of a new variant. In addition, low penetrant variants may affect the expression of a disease associated genetic variant. Therefore, there is still lack of enough evidence to offer a useful population screening [11,12].

## 4. Strategy in Consanguineous Families

In some cultures, building a family with a relative is a tradition and this increases the accumulation of rare pathogenic variants in the extended family, especially in areas with high incidence of certain disorders. Moreover, this also increases the risk for an individual to carry more than one gene with a deleterious variant. 

Consanguinity increases the risk for a recessive genetic disorder as the probability for a first cousin to carry any genetic variant, pathogenic or not, is 1/8, 12.5 percent. This means that even for ultrarare disorder there are increased risk. For example, if the incidence of a rare disorder is 1/90,000 in the population, the risk by chance for an affected child to first cousins are 1/4800 i.e., about 20 times increased. If you happen to know that one of the cousins is a carrier, the risk increases to 1/600 for an affected child. i.e., 150 times higher than for two unrelated persons from the same population. A targeted genetic test, consisting of the gene in question or sometimes just a specific pathogenic variant, can be offered to those who due to ethnicity have an increased risk. 

Testing for disorders with high incidence in specific populations have therefore been used for more than 40 years, e.g., thalassemia testing in Southern Europe and Middle East populations or testing for Tay-Sachs disease among the Ashkenazi Jewish population [13,14].

A genetic test, preferably offered to both partners, should be as broad as possible to cover as many situations as possible. Therefore, today whole exome or genome sequencing can be an option when filtering out common rare variants and be offered to a more general population with no high risk for specific pathogenic variants [15,16].

## 5. Panel Construction

With the recent advances in molecular genetic technology testing for hundreds or thousands of genetic pathogenic variants is possible and different companies and clinical laboratories have developed their own gene variant panels covering from some few disorders to several hundred variants, see examples on websites. 

https://www.europeanspermbank.com/en/services/screening-your-genes/facts-genexmatch (accessed on 10 September 2024).

https://qgenomics.com/service/qcarrier-test_en/ (accessed on 10 September 2024).

https://web.fulgentgenetics.com/reproductive/beacon-carrier-screening (accessed on 10 September 2024).

However, the patient information provided by the commercial providers may not be complete or neutral, instead rather often show a bias on the positive effect of performing a test [17]. 

It is challenging for the bioinformatician to interpret the data and together with a clinical geneticist filter out relevant pathogenic variants. Further, it requires qualified genetic counselling by a specialist to help the couple to understand the complexity of the test situation [18,19]. 

To construct a reliable test for PPGT some criteria must be fulfilled: Basically, only monogenic, highly penetrant autosomal or X-linked genetic variants causing severe disorders, including malformation syndromes, severe intellectual disability and non-curable disorders with early death, should be included in the test. Not many genes fulfil these criteria and they are usually among the most common of the recessive rare diseases with a prevalence in the population of more than 1/40,000, meaning that more than 1/100 in the population are carriers. However, how common a disorder is can vary among different populations. If your aim is to construct a broad fit-for-all gene panel, it need be very broad and necessarily include variants that in subpopulations will be very rare [20]. 

Carriership of more than 1/100 is sometimes used by genetic professionals to set a reference value for carriership for testing for recessive genetic disorders. Basically, if the number of carriers for pathogenic recessive variants in a gene is higher than one percent it is counted as a common variant and inclusion in a test panel is recommended. Based on this definition, carrier frequency in any larger subpopulation should be one percent or higher. This means, based on mendelian calculation, that the disease incidence is higher than 1/40,000. It is considered that in these common rare disorders and syndromes the gene-symptom correlation is well stablished. Thus, The American College of Genetics and Genomics recommend testing of a panel of 86 autosomal and 16 X-linked recessive genes. By doing so their intention is that any larger population in the US should avoid feeling discriminated [7]. 

Based on these data Schmidtke and Krawczak calculated that the probability to carry any of these 86 autosomal recessive variants were 69.6% for single individuals and 3.6% for couples, but that the risk differs between ethnic groups [21,22]. It should be noted that these figures assume of a carrier frequency, not the possibility to detect a variant by a genetic test. However, it is calculated that in the target population, more than 60 percent of the genetic load for autosomal recessive disorders is covered. Based on Orphanet data, it was recently found that the overall prevalence of recessive disorders were 3.5–5.9 percent based on estimated point prevalence of 5304 diseases [23]. Further, 149 disorders were estimated to be responsible for almost 80 percent of the disease burden which fits well with the estimates of Schmidtke and Krawzcak [22]. 

However, this approach is still limiting the panel to a certain population and its fitness for example for a Scandinavian or Nordic population where you find another mix of disease associated pathogenic variants have to be evaluated. It has also been argued that to be non-discriminating, panels should be as large as possible even with the problems of interpretation that may arise [21].

## 6. Clinical Utility, Validity, and Analytical Validity 

Clinical validity, clinical utility, and analytical utility are critical concepts in evaluating the effectiveness and appropriateness of diagnostic tests.

Clinical validity refers to the ability of a diagnostic test to accurately identify or predict the presence or absence of a particular disease or condition. Clinical utility relates to the practical value of a diagnostic test by leading to an improved health outcome for the patient. Finally, analytical utility refers to the technical performance of a diagnostic test in terms of its precision, accuracy, reliability, reproducibility, and robustness across different laboratory settings.

A stable and reliable test for a specific gene variant or set of variants should fulfil these requirements. However, for many genes we have only scarce information making the possibilities to perform proper evaluation difficult. Therefore, a laboratory report should include information on what is known about clinical and analytical validity so that clinical utility can be estimated in an as reliable way as possible. Moreover, in the situation of PPGT there is no internal control, i.e., no person with the disorder associated with this specific variant, which makes it even more important to only address results obtained by a test method that fulfils these criteria [6,24].

## 7. Polygenic Risk Score

Genetic factors play a significant role in the development of many complex diseases, including cardiovascular disorders, cancer, and psychiatric conditions. Polygenic risk scores (PRS) have emerged as powerful tools in genetic research, offering insights into the complex interaction between genetic variation and disease susceptibility. By integrating information from multiple genetic variants across the genome, PRS enable personalized risk assessment for various health conditions.

The following criteria defines polygenic risk score:

- PRS are computed by aggregating the effects of multiple genetic variants. These variants, known as single nucleotide polymorphisms (SNPs), are weighted based on their effect sizes, reflecting the magnitude of their association with the phenotype of interest.
- PRS is calculated using statistical models that evaluate the association between genetic variants and specific traits or diseases, often derived from large genome-wide association studies (GWAS).
- The score is designed to predict the likelihood of developing certain conditions (e.g., heart disease, diabetes, or mental health disorders) based on genetic factors, alongside environmental influences.
- PRS is not definitive; it provides probabilities rather than certainties. Environmental factors, lifestyle choices, and non-genetic influences also play significant roles in disease development.

Despite their potential, polygenic risk scores face several limitations and challenges. These include the need for large sample sizes to identify robust genetic associations, potential biases due to population stratification and ancestry differences, and limited generalisability across diverse populations.

So far, the benefit for the patient or the health care system is not validated good enough, and polygenic risk score should definitely not be used in the context of PPGT [25,26].

## 8. Advantages of PPGT 

Knowledge of the “genetic profile” empowers individuals and couples to make informed reproductive decisions regarding family planning. The chance for a healthy child is 75 percent in each pregnancy if both are carriers of a recessive pathogenic variants in the same gene. Parents-to-be can after proper genetic counselling weigh the risks and benefits with a test and abstain from utilising reproductive technologies such as prenatal or preimplantation genetic testing. Studies have shown that the uptake varies depending on the life situation of the individuals or couples included [27,28,29].

By accepting an offer of PPGT before pregnancy, the couple can identify potential genetic risks for having an affected child. This allows for proactive strategies, including prenatal or preimplantation diagnoses as well as the possibilities of alternative reproductive options such as adoption or assisted reproduction with egg or sperm donor for couples at high risk having an affected child.

Moreover, identifying carriers, facilitating informed consent for PPGT, will affect the number of children born with these disorders making them even more rare. However, it will take hundreds of generations to affect the number of carriers in the population and decrease the number of couples at risk. 

## 9. Drawbacks of PPGT

Despite its benefits, PPGT is not without challenges and drawbacks: Offering genetic counselling to individuals or couples who have no previous knowledge about the disease associated with the pathogenic variant, either among a family member or themselves is more challenging as they have no perception of the disease in advance. Also, even if the selection of genes for a test is selective, for many genes there are phenotypic variation of the disease depending on expressivity and penetrance, where an existing family record may give help in discussing risks. 

Although carrier status for most recessive genetic disorders is without health issues, sometimes there are heterozygote manifestations. For example, the X-linked recessive disorder haemophilia may have manifestations in female carriers with increased risk for bleeding. Similarly, the FragileX syndrome may, in pre-mutation carrier women be associated with early menopause, and in males may cause ataxia in early senescence. 

Another complex issue is mosacism. By this is meant an individual who was developed from a single fertilized egg with two or more cell populations with distinct genotypes, but mostly with a normal phenotype. As after the fertilisation the zygote consists of a single cell. Thus, the different cell populations must have emerged during embryogenesis, and the earlier this event occurred, the more widespread in the body will the mosaicism be. It may or may not affect the individual but can sometimes be associated with specific disorders. The deviant clone may be found among the germ cells and thus be inherited in the zygote and result in a child with a presumed de novo phenotype and often only detected if it has a dominant genetic effect. The possibility to detect mosaicism with PPGT is limited [30].

Moreover, due to emotional implications when receiving results of a genetic test the ability to offer a sensitive communication has a crucial role in PPGT, requiring skilled professionals for the interpretation of test results. The counsellor needs to be able to provide accurate information and support the individual person or couple in making reproductive decisions. 

Further, the understanding and impact of figures as high or low risk is a subjective matter and can be taken up by the counselee differently depending both on how the risk figures are presented and personal references [31,32,33].

Geographic location, cultural barriers and financial resources may limit access and uptake of PPGT when offered. Thus, information and educational initiatives to lay people as well as health care professionals are needed to raise awareness of PPGT options as was the case for hereditary breast cancer in the 1990-ies. This information must be neutral stressing both benefits and limitations. 

Comprehensive education initiatives are needed to raise awareness of PPGT options, benefits, and limitations among healthcare providers and the general public. Efforts to enhance accessibility to PPGT services, particularly for underserved populations, are essential for equitable healthcare delivery.

The cost for PPGT is on the individual person or couple, who either pay out of pocket or via a private health insurance. Increasing socioeconomic inequalities and inequalities in access must be considered in health care plans. Those who gain risk knowledge via PPGT will probably also require same possibilities for prenatal service and be allowed to receive it on similar conditions as others. This might affect accessibility for both genetic counselling and prenatal testing. 

However, state funded offer of PPGT may create social pressure or obligation to participate not to give birth to an affected child. Similar concerns have been highlighted earlier when introducing new technology for prenatal diagnosis. PPGT could lead to unforeseen consequences for society, or the individual born with a “preventable” genetic disease [34].

National health care programmes offering prenatal and/or preimplantation genetic testing are implemented in many countries worldwide [19], whereas programmes for offering PPGT are rarer and usually offered as part of research projects. 

In 2018, the Australian Government decided to fund a large national research study named Mackenzie’s Mission—the Australian Reproductive Genetic Carrier Screening Project. The number of tested genes in this project comprised 1281 variants associated with around 750 genetic conditions. The project recruited 8350 couples and was closed December 2022. Results of the study is expected to be published very soon [35].

In 2020 the Royal College of Pathologists of Australasia applied to the Medical Services Advisory Committee that all women and their partners in early pregnancy or planning a pregnancy should be eligible for Medicare-funded screening for three genetic conditions: cystic fibrosis, spinal muscular atrophy, and fragile X syndrome. Since 1 November 2023 this test is available covered by national health insurance and distributed via the primary health care providers. However, no extra resources have been given to genetic service raising questions of problems for genetic counselling. Thus, Australia is probably the first country worldwide to introduce such programme, although several countries have programmes for specific ethnic groups [36].

PPGT raises also a new approach to ethical considerations regarding autonomy, privacy, and the potential for genetic discrimination. Healthcare providers must navigate among these ethical complexities while upholding principles of patient confidentiality and informed consent.

## 10. The Importance of Genetic Counselling

Genetic counselling plays a crucial role in genetic testing. A genetic counsellor can in a pre- test counselling session bring up all those issues facilitating the road for the individuals or couples to be able to give an informed pre-test consent. Written or web-based information of high quality is a necessary tool in the consent process. The consent should be a signed document included in the patient files revealing what the counselees have consented for. Post-test counselling should provide accurate information, interpret test results, support in reproductive decisions, and offer psychosocial follow up during the process. It is necessary for each unit who aim at offer PPGT to develop a consistent information tool that can be used to the patients and also used for evaluation of the counselling session. 

The US National Society of Genetic Counselors have recently issued practical guidelines for reproductive risk assessment related to PPGT. Those, as well as the European Society for Human Genetics guidelines on direct-to-consumer genetic testing offer valuable reading and as a template for local, or preferably national guidelines [6,10].

## 11. Ethical and Societal Considerations

PCGT provides a possibility to empower couples to make more in depth informed reproductive choices. However, the introduction of PPGT also raises several ethical considerations based on common ethical principles, such as autonomy and justice. If offered through public healthcare a discussion about how to prioritise are necessary, as are issues concerning human dignity, solidarity, and cost-effectiveness. The characteristic of genetic testing raises complex issues around consent procedures, utility, and ownership of data

Autonomy is a key concept in genetic counseling. It involves the right to self-determination over one’s own life and own actions as long as it does not violate the right of self-determination of others. Autonomy also includes the right to choose to know or abstain the right to undergo medical treatment based on an informed consent, and the right to confidentiality [9]. Further, there is a consensus that carrier screening strengthens reproductive autonomy enabling more informed reproductive choices based on personal values and preferences of a couple [2].

The introduction of PPGT may affect family planning and reproductive decisions, making it possible for a couple to avoid a firstborn with a genetic disability, thus reducing the burden of care for an affected child [37].

Both the individual´s and the couple’s reproductive autonomy is enhanced [38] but new dilemmas might rise as joint autonomous decision might be required from the couple depending how the test results are disseminated [9,39,40].

The single unit approach for receiving test results aims at informing the partners jointly about whether they are a carrier couple or not. In case of a discordant outcome, individual carrier status is not reported. This deprives a possible carrier of the option of informing his or her relatives affecting reproductive autonomy of other family members, and using this information in an eventual future relation with another partner [40].

Another aspect is that carriers might also have some symptoms, for instance breast cancer risk in female ataxia telangiectasia carriers, demanding medical attention. If only couples are informed this risk will not be disseminated to the carrier or other relatives and affect their autonomy and right to choose to know [38] and to get right medical attention [41]. A shared relational decision might also be complex as moral and ethical values might differ both between the two partners, but also between the two different families of the couple, including political and socioethical s as well as values in different societies [39].

The alternative approach is viewing the couple as two individuals testing simultaneously and then informing about all individual outcomes. This leads to the identification of discordant couples, raising the demand for genetic counselling and cascade screening of relatives. Being identified as a carrier might have a negative psychological impact on well-informed testee [41,42], as carrier status has shown to increase stress, but on the other hand, other studies have shown that the influence on stress is not significant [43]. 

The negative impact of knowing a carrier status seems also to depend on how the screening programme is offered and how the information is given [40]. Proper education about the meaning of being a carrier is crucial and knowledge is instrumental in influencing participation. This should also include the fact that in terms of carrier status for recessive disease, we are all carriers [39].

A need for genetic counselling is thus obvious, both pre- and post-testing. However, a limiting factor is that it is not always available. Autonomy requires voluntary participation and informed consent which builds on educative parts as to benefits and limitations. Studies have shown that it is important to provide understandable, balanced information and education to the general public regarding the concepts of inheritance, when presenting the option of carrier screening [29].

Socioeconomic status affects the uptake of reproductive carrier screening [44,45,46,47], especially if PPGT is only offered by private providers and therefore only accessible for those that can afford to pay out-of-pocket. A public healthcare offer might, on the other hand, give the message that it is recommended or mandatory. With time, the societal pressure might increase, affecting autonomous decision making, with a risk for discrimination based on the decision not to take a test thus avoiding the birth of a severely affected child. It also has been argued that it is a medicalisation of a normal pregnancy [48]. Further, it could create stigma for those with a genetic disease, as well as for couples not joining a PPGT programme [38].

Preconception carrier screening thus moves towards becoming a moral duty and a pressure to take as showing that you are a responsible parent [48]. When deciding whether to introduce a programme or not, it needs to be considered if patient autonomy or societal preventions is the goal [48]. A decision involves many aspects to consider; medical, moral, psychological, and social. It is complex and therefore political and socioethical reflections are an essential concern in introducing PPGT [38].

Couple testing is influenced by the fact that knowledge in the society about the possibilities of testing is limited. Also, efforts to enhance accessibility to PPGT service and to raise awareness are essential for equitable health care services as PPGT is relatively unknown for most future parents. Also, here socioeconomic factors influence accessibility, and who will know to ask for and receive PPGT [44,45,46].

PPGT enables screening of a large number of recessive conditions in the same panel of known genetic variants, regardless of ancestry and geographic origin of users which also enhance health equity [49]. However, the selection of genetic variants to such a panel may not include rare disorders that may be more common in a subpopulation where founder mutations have locally a high prevalence [50]. Basic knowledge of the pattern of recessive variants in the population to test facilitates a high-quality panel.

Accessibility to PPGT may also be limited due to legal and cultural constraints. Countries have variable legal limitations to restrict access to service. Cultural influence, including religious beliefs, will also put limits on what is acceptable for individuals. This could lead to a discrimination for those who want to have a test. Distance to service centres may add to restricted access although it should be possible to overcome with access to web platforms for information and pre- and pos-test counselling, and surface or courier mail service for transporting tests to the testing centre.

Understanding patient preferences is important to reach the ethical goals of respect for and to support the patient. What is the will of parents-to-be? Would a governmental offer of PPGT offer stress, and press couples to accept a test or would it be supportive? In an Australian study 2/3 were positive [51], whereas studies in Sweden and the Netherlands showed lower interest [29,52].

Many of the participants in these studies believe that PPGT is of interest only for those with a positive family history of a hereditary disorder. However, overall, participants of reproductive age in the general Dutch population showed a positive attitude to predictive genetic testing, especially among young participants [29].

Moreover, the more evident treatment options that were available for the tested diseases, the more positive was the attitude of the participants for a predictive genetic test and supporting the equal possibility for PPGT as a tool for reproductive autonomy for everybody and not only for those without economic constraints [29].

In the study, the most frequently selected argument in favor of PPGT was the possibility to spare a child from a life with a severe hereditary disorder. The reason most often mentioned for not to participate, was that participants reported not having a hereditary disorder in the family. The majority preferred receiving individual test results above a couple-based disclosure method in which participants receive the carrier status results only when they are a carrier couple of the same disorder [29].

For lay people, risk information, including statistical numeric data, is perceived as highly normatively charged, often as an emotionally significant threat. Thus, it seems necessary to provide lay people with a deeper understanding of risk information and of the limitations of genetic knowledge with respect to one’s own health responsibility [53].

Genetic information also has emotional impact and ethical and moral issues that people have different views about, and relevant testing has results have become more complicated to decipher and being generated from more sources than ever before. This complexity increases the risk that genetic information will be misinterpreted or used inappropriately with harmful effects [54].

Choices between medical courses of actions may give patients a sense of safety and control and makes it easier to accept difficult medical information [55,56].

This brings up the importance of education, both for the health-care professionals and the public as limited access to PPGT can also be due to the level of education of the individual and professionals. Genetic literacy, knowledge about existence of the test and the possibilities to pay for service are constraints for accessibility to the test. In the coming years it will be very important to focus more on providing continuous high-quality information to the general public in order to improve genetic literacy, to reduce misconceptions and to manage expectations [51,57,58].

Another issue is why we offer testing for aneuploidies, but not PPGT as the chance of conceiving a child affected by an autosomal recessive disorder are closely comparable to that an aneuploidy [59,60,61]. On the other hand, does the offer of PPGT for a limited number of pathogenic variants substantially lower the risk for an affected child?

The regulatory landscape around PPGT pose similar challenges as in other situations for genetic testing; the consent process, data protection and access to pre- and post-test genetic counselling in a language understandable for the tested individuals. Harmonization of the regulatory system between national jurisdictions, e.g., within the European union should be strived for to uphold good clinical practice for PPGT. Knowledge is instrumental in influencing participation. Having good genetic knowledge may not be enough to understand core concepts of PPGT and may impact informed decision-making. Continuous education of health professionals and the community is crucial to reduce misconceptions of PPGT [51].

PPGT make embryo testing possible, and an increased use of this technique will affect costs for health care. However, it will also reduce the number of affected children born, thus reducing the burden of disease for the family and the society. However, the objective for PPGT should not be saving money, instead it should increase reproductive autonomy and equity and decrease suffering. Based on screened patients predominantly with private cost coverage, preconception PPGT is predicted to reduce the burden of Mendelian disease in a cost-effective manner compared with more minimal screening [62].

If PPGT is offered, the society should provide resources for genetic counselling, similar to what is offered for prenatal diagnostics and PPGT. If abortion is not an option, due to ethical and moral values in the society, but prenatal or embryo genetic testing nevertheless is offered, the results should be delivered with adequate counselling. This may facilitate both medical and psychological preparedness for giving birth to an affected child. However, the effect on other healthcare needs must be evaluated as prioritisation is necessary in a public health care system [8].

As the field of genetics and genomics has diversified over the past decade, the concept of non-directiveness has come under increasing pressure. While new models of genetic counselling practice are emerging as the complexity and number of ethical considerations relevant to genetic counseling has increased [63,64], most of them continue to emphasize some version of patient autonomy as the core principle. To complement prior work that has attempted to move away from non-directiveness, it has been argued that genetic counsellors should embrace a more explicit commitment to the principles of beneficence and non-maleficence, in addition to a broader understanding of both individual autonomy and the relational variables that influence the counselling process [54].

They argue that genetic counsellors should consider when it would be ethically acceptable, or even desirable, to make recommendations to patients in certain areas of their work. Their main premise is that genetic counsellors should use professional judgment and evidence to assess whether genetic testing might benefit a patient irrespective of her views about it. Non-maleficence, a principle of harm minimization, implies that genetic counsellors use expertise and evidence to prevent genetic testing and information from being used harmfully, where possible. Further, the complexity increases the risk that genetic information will be misinterpreted or used inappropriately with harmful effects [64]. The goal to enable autonomous reproductive choice can be challenging for both the counselee and the genetic counsellor [2,59].

In summary: efforts to enhance accessibility to PPGT service is needed to raise awareness and is essential for equitable health care services. Genetic literacy and knowledge about existence of the test and the possibilities to pay for service are probably constraints for accessibility to the PPGT. Underserved population need special attention because there could be language and/or educational constraints. However, as long as service is not included in health care programmes or otherwise subsidised inequalities will exist in access.

## 12. Some Points to Consider for Those Who Intend to Offer PPGT Service

To optimise the use of PPGT, the following suggestions for practical guidance can be considered:

- The provider should ensure that the counselees fulfil the requirements for PPGT genetic counselling under standard circumstances. Those families in which consanguinity is suspected, should be counselled separately according to relevant protocols, and considered to be offered testing outside a general PPGT programme.
- Genetic counselling should be integrated in the PPGT process to provide individuals with comprehensive support and guidance.
- Counselling sessions should address the emotional, psychological, and ethical aspects of genetic testing, empowering individuals to make informed reproductive decisions aligned with their values and preferences.
- Pre-and post-test counselling should be given by a qualified counsellor, medical specialist in clinical genetics, or other health care professional with skills to guide the counselee.
- The patient should have the possibility to opt out from the process at any time and allowed to re-enter at any time.
- The size and content of the gene panel should be discussed with the counselee so that limitations of the test are without doubt.
- The post-test counselling is most important in those cases where the couple has been found to have a risk for a child with a genetic disorder. The counselling should focus on the actual disorder and, if possible, also include contact with a specialist of the particular disorder.
- The governmental health care organisation should be encouraged to develop a panel that fits with the actual population and wide enough to include also disorders common in minority populations in the area.
- Even if provided on a pay-for-service basis, PPGT should be integrated into existing health care frameworks including the different stake holders, primary care providers, obstetricians/gynaecologists, paediatricians and others needed for care.

## 13. Conclusions

PPGT is still in its bud, but will most likely in the future, be a valuable new tool in reproductive healthcare, offering individuals the opportunity to assess their risk of transmitting genetic disorders to future generations. Further, PPGT offers new challenges related to ethical considerations, genetic counselling, and accessibility, it also holds a possibility to reduce the burden of hereditary disorders for the individual family. By addressing these challenges and implementing practical professional and legal guidance, PPGT can be effectively used to enhance reproductive outcomes and promote healthier pregnancies.
